# Sphingosine kinase 1-interacting protein is a novel regulator of glucose-stimulated insulin secretion

**DOI:** 10.1038/s41598-017-00900-7

**Published:** 2017-04-10

**Authors:** Yu Wang, Shin-ichi Harashima, Yanyan Liu, Ryota Usui, Nobuya Inagaki

**Affiliations:** Department of Diabetes, Endocrinology and Nutrition, Graduate School of Medicine, Kyoto University, Kyoto, 606-8507 Japan

## Abstract

Glucose-stimulated insulin secretion (GSIS) is essential in keeping blood glucose levels within normal range. GSIS is impaired in type 2 diabetes, and its recovery is crucial in treatment of the disease. We find here that sphingosine kinase 1-interacting protein (SKIP, also called Sphkap) is highly expressed in pancreatic β-cells but not in α-cells. Intraperitoneal glucose tolerance test showed that plasma glucose levels were decreased and insulin levels were increased in SKIP^−/−^ mice compared to SKIP^+/+^ mice, but exendin-4-enhanced insulin secretion was masked. GSIS was amplified more in SKIP^−/−^ but exendin-4-enhanced insulin secretion was masked compared to that in SKIP^+/+^ islets. The ATP and cAMP content were similarly increased in SKIP^+/+^ and SKIP^−/−^ islets; depolarization-evoked, PKA and cAMP-mediated insulin secretion were not affected. Inhibition of PDE activity equally augmented GSIS in SKIP^+/+^ and SKIP^−/−^ islets. These results indicate that SKIP modulates GSIS by a pathway distinct from that of cAMP-, PDE- and sphingosine kinase-dependent pathways.

## Introduction

Glucose is the most important physiological secretagogue of insulin secretion. Intracellular uptake and metabolism of glucose are the essential features of glucose-stimulated insulin secretion (GSIS)^1^. Dual, hierarchical control of insulin secretion by glucose is proposed: the triggering pathway and the metabolic amplifying pathway^2, 3^. The increase of the ATP: ADP ratio by glucose inhibits ATP-sensitive K^+^ (K_ATP_) channels, followed by depolarization of the β-cell membrane, opening of the voltage-dependent Ca^2+^ channels (VDCCs), and elevation of influx of extracellular Ca^2+^, which activates insulin granules exocytosis^3–7^. This K_ATP_ channel-dependent triggering pathway is particularly important in the first, acute phase of GSIS. The metabolic amplifying pathway, originally referred to as the K_ATP_ channel-independent pathway, contributes similarly to both the first and the second phase of GSIS^8^. Glucose increases insulin secretion from islets lacking K_ATP_ channels, partially contributing to amplification of the triggering action of Ca^2+^ ^9^. The non-electrical effects of glucose on amplification of GSIS depend on the metabolism of glucose^10^. Several candidates have been proposed in the metabolic amplifying pathway: ATP^11^, NADPH^12–14^, AMP-activated protein kinase^15^, SENP1^16, 17^, and S-AMP^18^. However, the mechanisms of the process are not fully understood.

Neurohormonal amplifying pathways also have a major role in enhancement of GSIS. The two incretins, gastric inhibitory polypeptide (GIP) and glucagon-like peptide 1 (GLP-1), potentiate GSIS by activation of 3′-5′-cyclic adenosine monophosphate (cAMP) signaling^19, 20^; elevation of the cAMP concentration acts through both a protein kinase A (PKA)-dependent and a PKA-independent mechanism^21, 22^, the latter involving the alternative cAMP sensor Epac2^23–25^.

Recently, we found that SKIP (also called Sphkap) was highly expressed in insulinoma cells, but not in other cell lines. Initially, SKIP was identified as a sphingosine kinase (SPHK) interacting protein in the brain of mice that inhibited SPHK activity *in vitro* ^26^. SKIP also was reported to be an A-kinase anchoring protein (AKAP) that binds to PKA regulatory subunit I (PKARI) in the heart^27, 28^, and phosphorylates the inner mitochondrial membrane protein ChChd3 (coiled-coil-helix-coiled-coli-helix domain containing 3) via cAMP-mediated signals^29^. AKAPs are a large group of proteins that anchor PKA to the membrane and cellular organelles. So far, over 70 molecules have been identified in the AKAP family^30, 31^. However, the involvement of SKIP in insulin secretion is still unknown.

We therefore examined the role of SKIP in insulin secretion. We show here that SKIP is highly expressed in pancreatic β-cells but not in α-cells and that depletion of SKIP amplifies GSIS by a pathway distinct from the cAMP-, PDE- and sphingosine kinase-dependent pathways.

## Results

### SKIP is highly expressed in the islets

**Figure 1:**
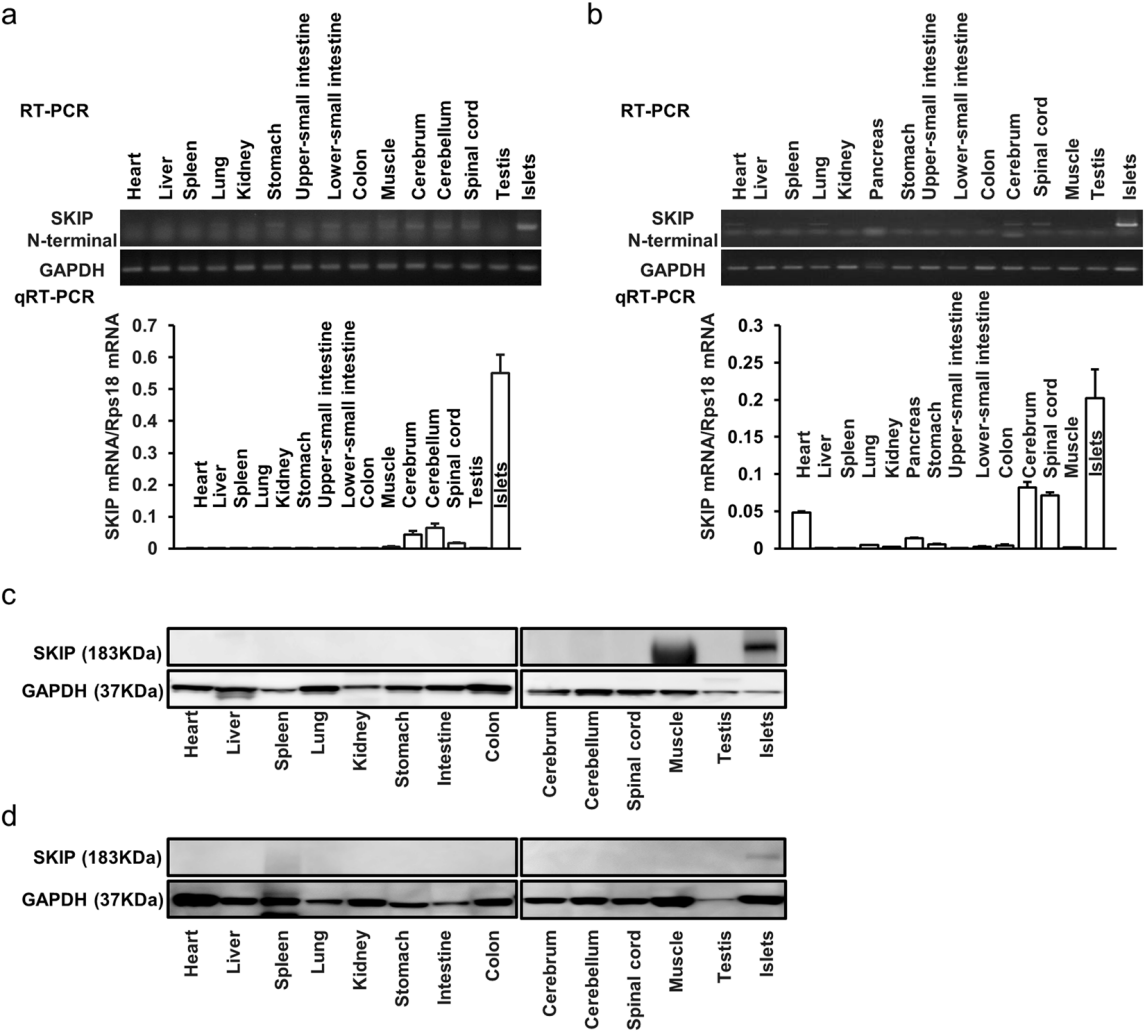
SKIP expression in various tissues. (**a**–**d**) mRNA expression of SKIP in several tissues from C57BL/6 mice (**a**) or Wistar rats (**b**) detected by RT-PCR and qRT-PCR; protein expression of SKIP in several tissues from C57BL/6 mice **(c**) or Wistar rats (**d**) detected by western blot with anti-mouse SKIP (**c**) and anti-rat SKIP antibody (**d**), respectively. The membrane was reprobed with anti-GAPDH antibody as control. All gels were run under the same experimental conditions. Uncropped images of blots/gels are shown in Supplemental Figures 2–4. (**a**–**d**) 12-week-old animals were used for experiments, n = 4.

We first examined SKIP expression in normal mouse and rat tissues and cells, as the molecule is highly expressed in insulinoma cells but not in other cell lines (Supplemental Table 1, Supplemental Figure 1). Reverse transcription PCR (RT-PCR) and quantitative real time PCR (qRT-PCR) revealed that SKIP mRNA was highly expressed in mouse (Fig. 1a) and rat islets (Fig. 1b) compared to that in other tissues. Western blot analysis also demonstrated that SKIP is strongly expressed in the islets but not in other tissues in mouse (Fig. 1c) and rat (Fig. 1d).

### Generation of SKIP knock-in (KI) mice

**Figure 2:**
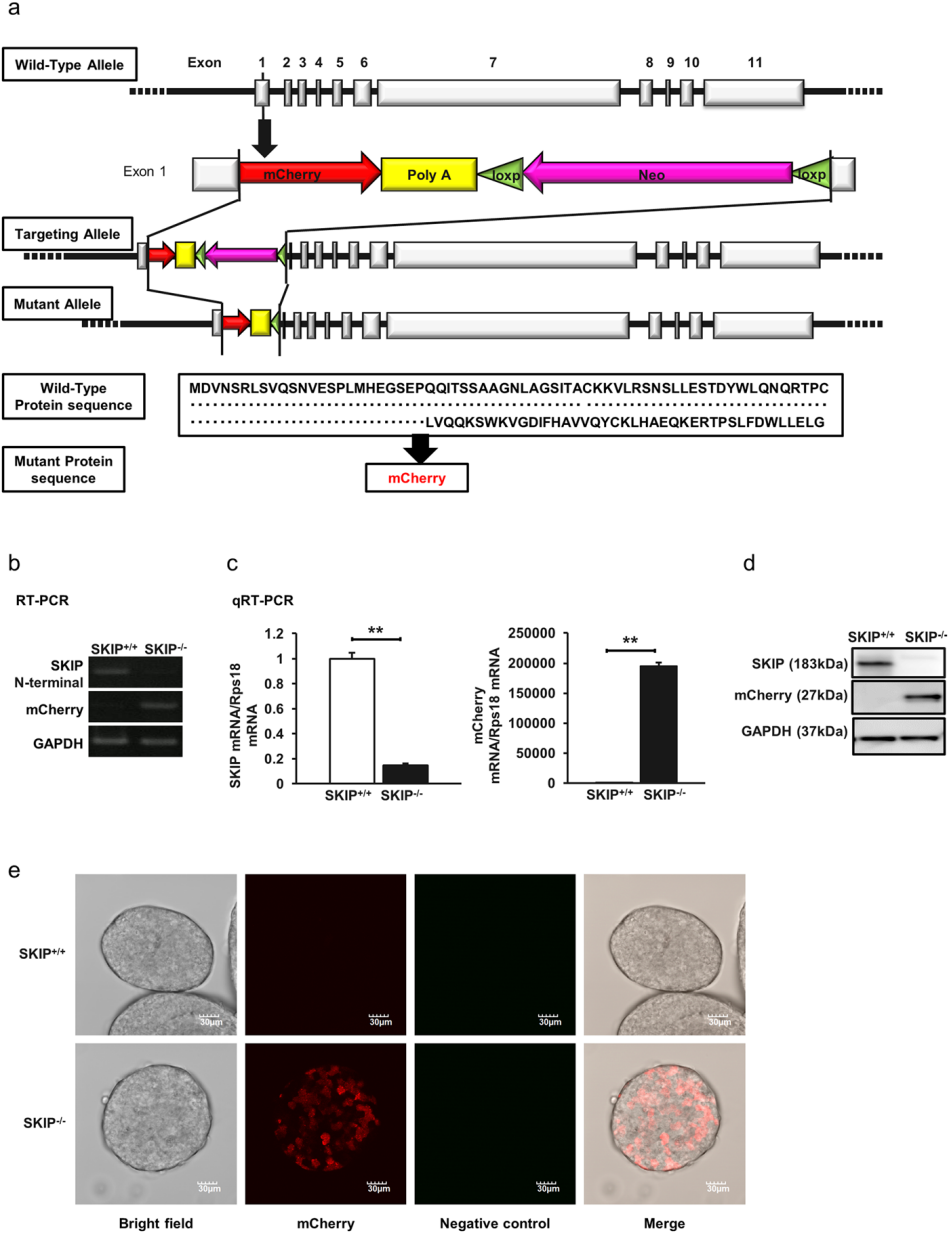
Generation of SKIP-mCherry knock-in (KI) mice. (**a**) Construct of SKIP-mCherry KI mice. mCherry-poly(A)-loxp-Neo-loxp was inserted into exon 1 of wild-type SKIP gene, and later, loxp-Neo was deleted to generate the mutant allele. In the mutant protein sequence, SKIP expression was deleted by mCherry expression. mRNA expression of SKIP and mCherry in isolated islets from homo SKIP-mCherry KI (SKIP^−/−^) mice and wild type (SKIP^+/+^) mice detected by RT-PCR (**b**) and qRT-PCR (**c**); data are expressed as average ± standard error of the mean (SEM). **p < 0.005 SKIP^−/−^ vs SKIP^+/+^, significance was determined by student’s t-test (**c**). (**d**) Protein expression of SKIP in isolated islets from SKIP^−/−^ mice and SKIP^+/+^ mice detected by western blot with anti-mouse SKIP antibody. The membrane was reprobed with anti-mCherry antibody, and reprobed with anti-GAPDH antibody as control. All gels were run under the same experimental conditions. Uncropped images of blots/gels are shown in Supplementary Figure 5. (**e**) Expression of mCherry in isolated islets from SKIP^−/−^ mice and SKIP^+/+^ mice. Living islets were observed by incubator two-photon excitation microscopy. Two-photon excitation was effected at 1040 nm, the fluorescence of mCherry and negative controls were measured at 575–650 nm and at 460–495 nm, respectively. (**b**–**e**) n = 4–6 mice per group and 3 samples per group, 12-week-old mice were used for experiments.

As SKIP is strongly expressed only in the islets, we generated SKIP-mCherry KI mice to evaluate the effects of SKIP on insulin secretion (Fig. 2a). Compared to SKIP^+/+^ mice islets, expression of the SKIP gene was absent and that of the mCherry gene was present in SKIP^−/−^ mice islets by RT-PCR (Fig. 2b). qRT-PCR showed that SKIP was decreased by 85% and that mCherry was detected only in the islets of SKIP^−/−^ mice (Fig. 2c). SKIP protein was not detected in SKIP^−/−^ mice (Fig. 2d). Incubator two-photon excitation microscopy showed that mCherry was expressed only in SKIP^−/−^ mice and not in SKIP^+/+^ mice (Fig. 2e). These results demonstrate that SKIP was successfully knocked down in the islets of SKIP-mCherry KI mice.

### SKIP is specifically expressed in pancreatic β-cells

**Figure 3:**
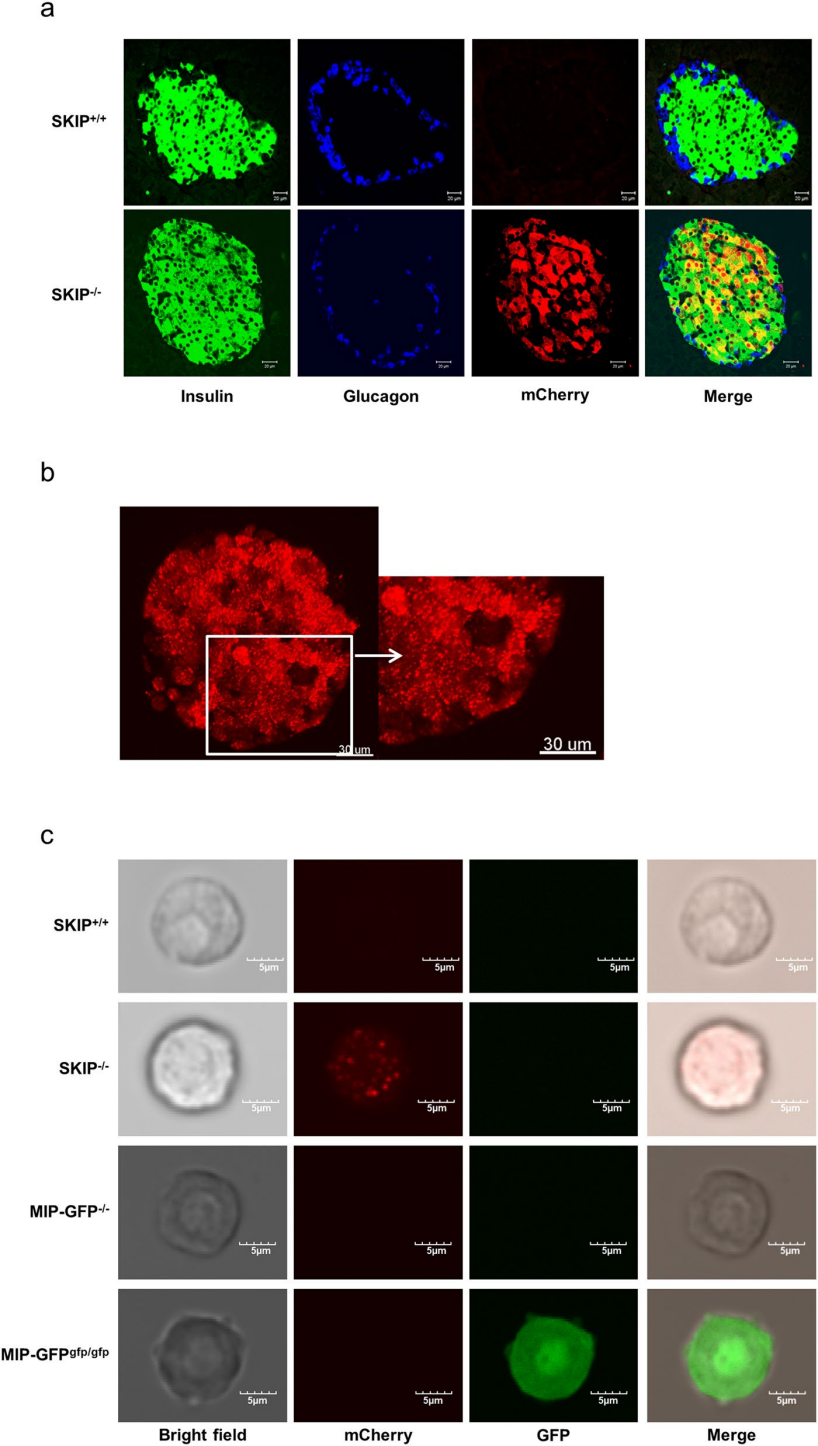
Localization of SKIP in the islets. (**a**) Immunohistochemical images in the pancreas from SKIP^−/−^mice and SKIP^+/+^ mice; green, anti-insulin; blue: anti-glucagon; and red, anti-mCherry. (**b**) 3D imaging by incubator two-photon microscopy in isolated islets from SKIP^−/−^ mice. (**c**) Incubator two-photon excitation microscopy images of living β-cells from SKIP^−/−^ (SKIP-mCherry KI) mice and MIP-GFP mice; left column, bright field; second column, mCherry; third column, GFP; right column, merged images. 12-week-old mice were used for the experiments, n = 3.

We examined SKIP localization in the islets to clarify the mechanism of SKIP-modified insulin secretion. Immunohistochemical analysis also showed that mCherry was detected only in β-cells, and not in α-cells from SKIP^−/−^ mice (Fig. 3a). Three-D imaging by incubator two-photon microscopy revealed mCherry signals to be visible in isolated β-cells from SKIP^−/−^ mice (Fig. 3b). Incubator two-photon excitation microscopy also showed the mCherry signal to be strongly detected in a single β-cell of SKIP^−/−^ mice, but not in SKIP^+/+^ mice, as displayed in a single β-cell of MIP-GFP mice^32, 33^ (Fig. 3c). These finding indicate that SKIP is specifically expressed in pancreatic β-cells.

### Glucose-stimulated insulin secretion is augmented by depletion of SKIP

**Figure 4:**
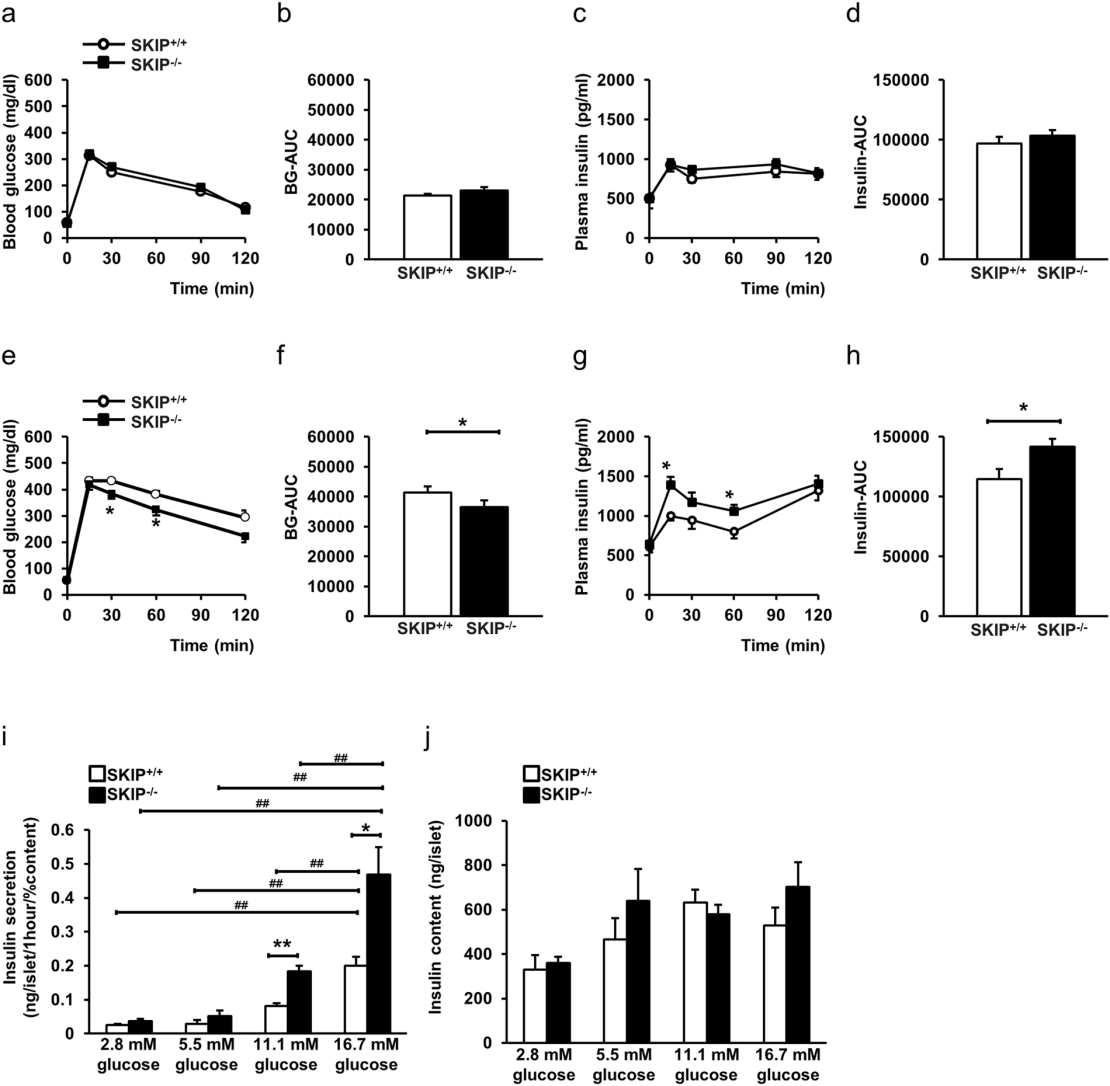
SKIP-regulated glucose-stimulated insulin secretion (GSIS). (**a**–**d**) Blood glucose levels (**a**), BG-AUC (**b**), plasma insulin levels (**c**) and insulin-AUC (**d**) during 1 g/kg body weight IpGTT. (**e**–**h**) Blood glucose levels (**e**), BG-AUC (**f**), plasma insulin levels (**g**) and insulin-AUC (**h**) during 2 g/kg body weight IpGTT. (**i**) GSIS in islets from SKIP^+/+^ mice and SKIP^−/−^ mice. Insulin secretion was measured at 2.8 mM, 5.5 mM, 11.1 mM, and 16.7 mM glucose. (**j**) Insulin content in the islets from SKIP^+/+^ mice and SKIP^−/−^ mice. (**a**–**j**) 12-week-old mice were used for the experiments. (**a**–**h**) n = 7–8 mice per group, *p < 0.05 vs SKIP^+/+^, significance was determined by student’s t-test. (**i**,**j**) n = 7–8 mice per group and 5–6 samples per group, with 10 islets per sample, *p < 0.05, **p < 0.005 vs SKIP^+/+^, ^#^p < 0.05, ^##^p < 0.005 for different glucose conditions in the same genotype mice of insulin secretion, significance was determined by one way ANOVA with Tukey test. All data are expressed as average ± SEM.

An IpGTT of 1 g glucose per kg body weight administration showed that blood glucose levels, blood glucose area under the curve (BG-AUC), plasma insulin levels and insulin area under the curve (insulin-AUC) did not differ in SKIP^+/+^ and SKIP^−/−^ mice (Fig. 4a–d). Under an IpGTT of 2 g glucose per kg body weight administration, blood glucose levels were significantly lower in SKIP^−/−^ mice than in SKIP^+/+^ mice at 30 min and 60 min (P < 0.05), and tended to be even lower at 120 min (P = 0.07) (Fig. 4e). BG-AUC also was significantly reduced by 15% (Fig. 4f). Plasma insulin levels were higher in SKIP^−/−^ mice compared to SKIP^+/+^ mice (Fig. 4g) at 15 and 60 min (P < 0.05), which resulted in an increase of insulin-AUC by 1.24-fold (Fig. 4h).

Next, insulin secretion was compared in the islets from SKIP^−/−^ mice and SKIP^+/+^ mice. At 2.8 mM and 5.5 mM glucose, insulin secretion was at a similar level in SKIP^−/−^ and SKIP^+/+^ mice (Fig. 4i). At 11.1 mM and 16.7 mM glucose, insulin secretion was about 2.5-fold higher, respectively, in SKIP^−/−^ mice than in SKIP^+/+^ mice (Fig. 4i). Interestingly, GSIS was amplified in islets from SKIP^−/−^ mice compared to SKIP^+/+^ mice. GSIS in SKIP^−/−^ mice was about 5.5-fold and 14.4-fold increased at 11.1 mM and 16.7 mM glucose, respectively, compared to that at 2.8 mM glucose (Fig. 4i, Supplemental Table 2). On the other hand, GSIS was about 3.2-fold and 7.3-fold increased at 11.1 mM and 16.7 mM glucose, respectively, compared to that at 2.8 mM glucose in SKIP^+/+^ mice (Fig. 4i, Supplemental Table 2). Insulin content was not altered among islets stimulated by the indicated glucose concentrations (Fig. 4j). These results indicate that depletion of SKIP augments GSIS under high glucose conditions.

### Exendin-4 (ex-4)-enhanced insulin secretion is absent by depletion of SKIP

**Figure 5:**
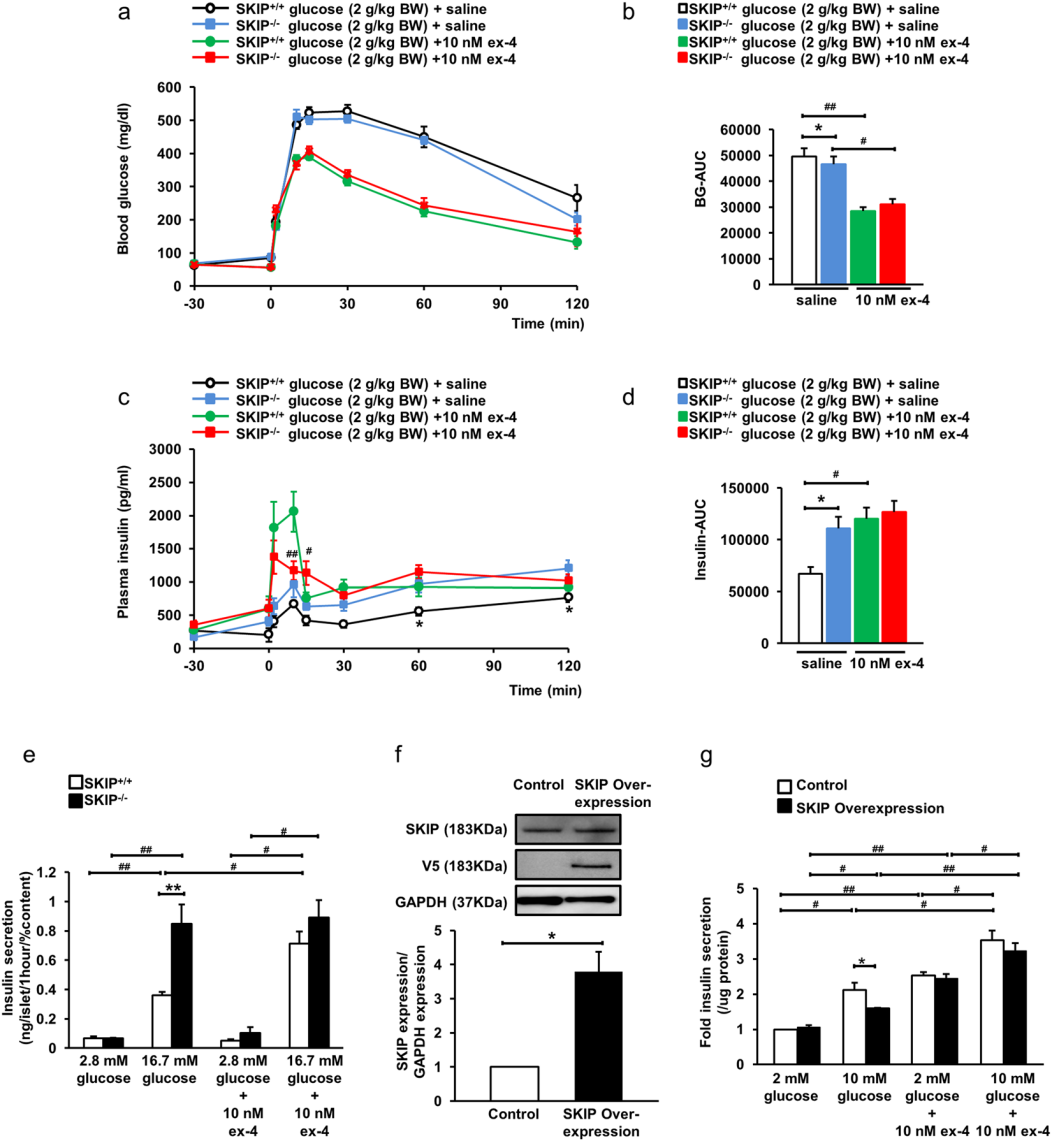
Exendin-4-enhanced Insulin secretion in SKIP^+/+^ mice and SKIP^−/−^ mice. (**a**–**d**) Blood glucose levels (**a**), BG-AUC (**b**), plasma insulin levels (**c**) and insulin-AUC (**d**) during 2 g/kg body weight IpGTT 30 min after administration of 10 nM ex-4. (**e**) Ex-4-induced insulin secretion in the islets from SKIP^+/+^ mice and SKIP^−/−^mice. Insulin secretion from isolated islets measured at 2.8 mM and 16.7 mM glucose with or without 10 nM ex-4. (**f**) Protein expression of SKIP in control and SKIP-overexpressed INS-1D cells after 48 h transfection detected by western blot with anti-rat SKIP antibody. The membrane was reprobed with anti-V5 antibody, and reprobed with anti-GAPDH antibody as control. All gels were run under the same experimental conditions. Uncropped images of blots/gels are shown in Supplemental Figure 6 (n = 3). (**g**) Insulin secretion in SKIP-overexpressed INS-1D cells measured at 2 mM and 10 mM glucose with or without 10 nM ex-4. (**a**–**e**) 12-week-old mice were used for the experiments. (**a**,**c**) ^#^p < 0.05 SKIP^−/−^ ex-4 vs SKIP^+/+^ ex-4. (**b**,**d**) *p < 0.05, **p < 0.005 SKIP^−/−^ saline vs SKIP^+/+^ saline, ^#^p < 0.05, ^##^p < 0.005 for different stimulated conditions in the same genotype mice. (**e**) n = 7–8 mice per group and 5–6 samples per group, with 10 islets per sample, *p < 0.05, **p < 0.005 vs SKIP^+/+^, ^#^p < 0.05, ^##^p < 0.005 for different stimulated conditions in the same genotype mice of insulin secretion. (**g**) Data from 4 experiments are shown, *p < 0.05 vs control, ^#^p < 0.05, ^##^p < 0.005 for different stimulated conditions in the same cells. All data are expressed as average ± SEM. Significance was determined by one way ANOVA with Tukey test (**a**–**e**,**g**) and student’s t-test (**f**).

Regarding the incretin effect, under an IpGTT of 2 g glucose per kg body weight in the presence of 10 nM ex-4, blood glucose levels at each point and the BG-AUC level in SKIP^+/+^ mice and SKIP^−/−^ mice did not differ (Fig. 5a,b). On the other hand, the plasma insulin levels in the presence of 10 nM ex-4 were significantly lower at 10 min and 15 min (P < 0.05) in SKIP^−/−^ mice than in SKIP^+/+^ mice (Fig. 5c), while insulin-AUC was similar in SKIP^−/−^ mice and SKIP^+/+^ mice at 0–120 min (Fig. 5d).

Regarding the role of SKIP in incretin-enhanced insulin secretion, at 2.8 mM glucose, 10 nM ex-4 did not increase GSIS in either SKIP^+/+^ or SKIP^−/−^ mice islets (Fig. 5e). At 16.7 mM glucose, GSIS in SKIP^+/+^ mice islets was increased 2.0 fold in the presence of 10 nM ex-4 compared to that in the absence of 10 nM ex-4 (Fig. 5e). On the other hand, GSIS at 16.7 mM glucose in the presence of 10 nM ex-4 was almost the same as that only at 16.7 mM glucose in SKIP^−/−^ mice islets (Fig. 5e).

Furthermore, full-length of rat SKIP with V5 tag was overexpressed in INS-1D cells to examine the role of SKIP in GSIS and ex-4-enhanced insulin secretion. SKIP expression was about 3 fold increased in SKIP-overexpressed INS-1D cells compared to that in control INS-1D cells (Fig. 5f). GSIS was decreased by 25% at 10 mM glucose in SKIP-overexpressed INS-1D cells compared to that in control INS-1D cells (Fig. 5g). However, ex-4-enhanced insulin secretion at 10 mM glucose in SKIP-overexpressed cells was almost the same as that in control cells (Fig. 5g).

These results indicate that GSIS under high glucose conditions may be almost fully amplified by the depletion of SKIP.

### The mechanism of regulation of glucose-stimulated insulin secretion by SKIP

**Figure 6:**
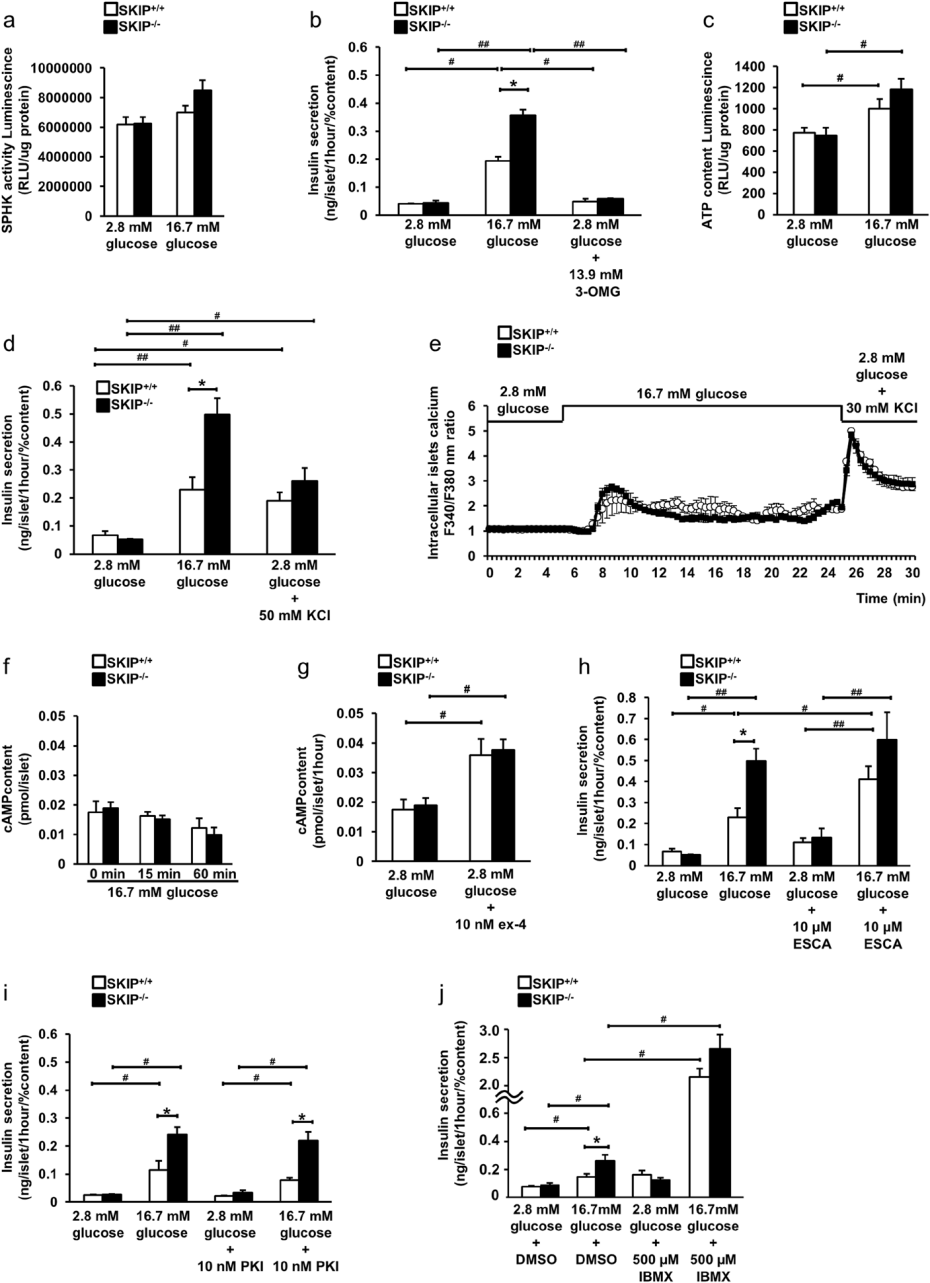
The mechanism of SKIP-involved insulin secretion. (**a**) Sphingosine kinase activity from isolated islets measured at 2.8 mM and 16.7 mM glucose. (**b**) Insulin secretion measured at 2.8 mM, 16.7 mM glucose and 2.8 mM with 13.9 mM non-metabolic glucose (3-OMG). (**c**) ATP content from isolated islets measured at 2.8 mM and 16.7 mM glucose. (**d**) Potassium-evoked insulin secretion in isolated islets from SKIP^−/−^ mice. Insulin secretion measured at 2.8 mM, 16.7 mM glucose and 2.8 mM with 50 mM KCl. (**e**) Calcium influx measured in islets from SKIP^+/+^ and SKIP^−/−^ mice. (**f**) cAMP content in isolated islets from SKIP^+/+^ and SKIP^−/−^ mice stimulated at 16.7 mM glucose for 0 min, 15 min, 60 min. (**g**) cAMP content in isolated islets from SKIP^+/+^ and SKIP^−/−^ mice at 2.8 mM glucose with or without 10 nM ex-4. (**h**) Epac2 selective activator (ESCA)-stimulated insulin secretion in islets from SKIP^+/+^ and SKIP^−/−^ mice. Insulin secretion measured at 2.8 mM or 16.7 mM glucose with or without 10 μM ESCA in the isolated islets. (**i**) PKA-dependent insulin secretion in islets from SKIP^+/+^ and SKIP^−/−^ mice. Insulin secretion from isolated islets measured at 2.8 mM or 16.7 mM glucose with or without 10 nM PKI. (**j**) GSIS treated with 3-isobutyl-1-metylxantine (IBMX) in islets from SKIP^+/+^ and SKIP^−/−^ mice. Insulin secretion was measured at 2.8 mM or 16.7 mM glucose with or without 500 μM IBMX in the isolated islets. (**a**–**j**) 12-week-old mice were used for the experiments. *p < 0.05, **p < 0.005 vs SKIP^+/+^, ^#^p < 0.05, ^##^p < 0.005 for different stimulated conditions in the same genotype mice of insulin secretion. (**a**–**d**, **h**–**j**) n = 7–8 mice per group and 5–6 samples per group, 10 islets per sample. (**e**) n = 3 mice per group and 3 samples per group, 12–20 islets per sample. (**f**,**g**) n = 6–7 mice per group and 5–6 samples, 25 islets per group. Data are expressed as average ± SEM. Significance was determined by one way ANOVA with Tukey test (**a**–**d**, **f**–**j**) and student’s t-test (**e**).

The detailed mechanism involved in SKIP-regulated GSIS was then examined. SPHK activity was similar in SKIP^−/−^ mice and SKIP^+/+^ mice islets at 2.8 mM and 16.7 mM glucose (Fig. 6a). Neither SKIP^−/−^ mice nor SKIP^+/+^ mice islets showed an increase in glucose-stimulated insulin secretion in the presence of non-metabolic glucose (3-OMG) (Fig. 6b). ATP content was equally increased in islets of both SKIP^+/+^ mice and SKIP^−/−^ mice, but it did not differ between the islets at 2.8 mM and 16.7 mM glucose (Fig. 6c). Potassium-induced insulin secretion also was found not to differ between the islets of SKIP^+/+^ mice and SKIP^−/−^ mice (Fig. 6d). The intracellular calcium concentration did not differ by stimulation with 16.7 mM glucose or 30 mM KCl between the groups of islets from SKIP^+/+^ mice and SKIP^−/−^ mice (Fig. 6e). The cAMP content was similar between the islets from SKIP^+/+^ mice and SKIP^−/−^ mice stimulated with 16.7 mM glucose for 0 min, 15 min and 60 min (Fig. 6f). cAMP content did not differ at 2.8 mM glucose levels, and was equally increased by 10 nM ex-4 in islets of both SKIP^+/+^ mice and SKIP^−/−^ mice (Fig. 6g). In addition, cAMP signaling did not affect enhancement of GSIS in SKIP^−/−^ mice islets compared to SKIP^+/+^ mice islets, nor did the Epac2 selective activator ESCA enhance GSIS (Fig. 6h), and the protein kinase-A inhibitor PKI did not decrease GSIS (Fig. 6i). Furthermore, 500 µM 3-isobutyl-1-metylxantine (IBMX), a non-selective phosphodiesterase (PDE) inhibitor, promoted GSIS at 16.7 mM glucose, but the total amount of GSIS was not significantly different between SKIP^+/+^ and SKIP^−/−^ islets (Fig. 6j). The increment of insulin secretion by 16.7 mM glucose + IBMX compared to that by 16.7 mM glucose + DMSO also did not significantly differ between SKIP^+/+^ and SKIP^−/−^ islets (Fig. 6j).

## Discussion

We show here that SKIP is a novel protein expressed in pancreatic β-cells and that depletion of SKIP augments glucose-stimulated insulin secretion (GSIS). Glucose metabolism was necessary for amplification of GSIS, but it was not depolarization-evoked or cAMP-enhanced, and PDE-involved insulin secretion differed in the islets with or without the SKIP molecule. Although the detailed mechanism remains unknown, our results demonstrate that depletion of SKIP exaggerates GSIS under high glucose conditions.

Initially, SKIP was identified as a sphingosine kinase (SPHK) interacting protein in the brain of mice that inhibited SPHK activity *in vitro* ^26^. It was reported that SPHK increased insulin secretion due to an increase in sphingosine 1-phosphate (S1-P) levels by glucose in pancreatic β-cells^34^. However, our data show that SPHK activity did not differ between islets of SKIP^+/+^ mice and SKIP^−/−^ mice. SKIP also was reported to be an A-kinase anchoring protein (AKAP) that binds to PKA regulatory subunit I (PKARI) in the heart^27, 28^. Previous studies reported that disruption of PKA-AKAP interaction decreased only cAMP-mediated insulin secretion^35, 36^ and did not affect GSIS^37^. On the other hand, recent study on PKARI subunit-deleted mice showed a dramatic increase in GSIS^38^. Our data also demonstrate that depletion of SKIP augments GSIS under high glucose conditions. However, cAMP-mediated signals in GSIS were the same in SKIP^−/−^ mice islets as those in SKIP^+/+^ mice islets. cAMP content at low glucose and at high glucose did not differ in SKIP^+/+^ and SKIP^−/−^ mice islets. A PKA inhibitor and an Epac2 activator did not modify GSIS in SKIP^−/−^ mice islets. PDEs as regulators of intracellular cyclic nucleotide concentrations can trigger multiple cellular signaling events^39^. PDE3B, 5, 7, 8, 9, 10, and 11 are expressed in rodent and human islets^40–42^, and inhibition of PDE3B and PDE8 potentiates insulin secretion^43^. However, in our study, a general PDE inhibitor IBMX enhanced GSIS at 16.7 mM glucose, although there was no significant difference in insulin secretion levels between SKIP^+/+^ and SKIP^−/−^ islets. This evidence clearly indicates that another pathway distinct from the cAMP-, PDE- and sphingosine kinase-dependent pathways is related to augmentation of GSIS in depletion of SKIP.

GSIS is the principal process of insulin secretion^44^. Glucose is metabolized to pyruvate followed by an increase in the ATP concentration, closure of K_ATP_ channels, depolarization of the β-cell membrane, opening of the voltage-dependent Ca^2+^ channels (VDCCs), and promotion of Ca^2+^ influx^6^. This process is the main pathway of GSIS. However, ATP content and depolarization-evoked insulin secretion did not differ between SKIP^−/−^ mice and SKIP^+/+^ mice islets.

The metabolic amplifying pathway also is an important pathway for GSIS^2, 3^. Glucose was reported to increase insulin secretion from mouse islets lacking K_ATP_ channels, and the effect could be attributed partially to amplification of the triggering action of Ca^2+^. Although the mechanisms of the process are not yet clear, ATP, adenine nucleotides, and other messengers could be involved in the pathway. However, in our study, the ATP content and intracellular Ca^2+^ concentration did not differ between islets of SKIP^+/+^ mice and SKIP^−/−^ mice. Recently sentrin/SUMO-specific protease 1 (SENP1) and adenylosuccinate (S-AMP) signals were reported to be important for the metabolic amplifying pathway^18^. SENP1 knockout mice showed a decrease in GSIS without any changes in intracellular Ca^2+^ responses compared to wild type mice^17^. Addition of S-AMP to the interior of patch-clamped human β-cells was found to amplify exocytosis dependent on SENP1 expression^17, 18^. In our data, the amplification of GSIS in SKIP^−/−^ mice was distinct from K_ATP_ channels and cAMP-mediated signals, implying that the metabolic amplification pathway is somehow involved in SKIP-modulated insulin secretion.

Glutamate also is a signal linking glucose metabolism and incretin/cAMP action to amplify insulin secretion^45, 46^. Glucose increases cytosolic glutamate and the cAMP/PKA signal promotes glutamate transport into insulin-containing secretory granules, leading to amplified insulin secretion^46^. However, in SKIP^−/−^ islets, PKI did not affect insulin secretion despite the requirement of glucose metabolism in GSIS. Epac2A promotes insulin secretion of glutamate-containing insulin granules, but ESCA, an Epac2 selective activator, did not amplify GSIS in SKIP^−/−^ islets. This suggests that glutamate is not a major factor in the amplification of GSIS in SKIP^−/−^ islets.

In conclusion, SKIP is highly expressed in pancreatic β-cells but not in α-cells, and depletion of SKIP amplifies GSIS under high glucose conditions by a pathway distinct from the cAMP-, PDE- and sphingosine kinase-dependent pathways. Reduction of glucose sensitivity is pathognomonic of the β-cell defect in type 2 diabetes (T2DM)^47^. T2DM also is associated with reduced expression of key exocytosis proteins^48^, although this does not reduce depolarization-evoked exocytosis. In fact, GSIS in T2DM is more impaired than insulin secretion induced by sulfonylureas or high K^+^ *in vitro* ^49, 50^. These findings indicate that the functional defect of β-cells lies upstream in the stimulus-secretion pathway of intracellular Ca^2+^ elevation and the generation of electrical activity in T2DM. To develop novel drugs for treatment of the disease, it is essential to understand the detailed processes involved in the triggering pathway, the metabolic amplifying pathway, and the neurohormonal amplifying pathway of glucose-stimulated insulin secretion. Our present data suggest that modulation of SKIP expression in the β-cells may represent a novel target for clinical treatment of T2DM.

## Materials and Methods

### Generation of SKIP-mCherry knock-in mouse

We designed target vector constructs as short-mCherry-SV40polyA-loxP-Neo-loxP-long-DTA cassettes using mouse BAC clone (identification number RP23-401A20 or RP23-227N4). The F1 mice were mated with CAG-Cre mice to generate the F2 hetero mice not having the neomycin sequence, and then removed the Cre sequence by mating the F2 hetero mice with wild type mice to generate F3-SKIP^+/−^ mice hetero mutant mice.

### Animals

Animal care and procedures were approved by the Animal Care Committee of Kyoto University. The methods were carried out in accordance with the Animal Care Committee of Kyoto University. Twelve-week old male homo SKIP-mCherry knock-in (KI) mice (SKIP^−/−^) and wild type (SKIP^+/+^) littermates were used in all experiments. MIP-GFP knock-in mice were kindly provided by Dr. Minami Hara^32, 33^, and 12-week-old mice were used for experiments.

### Islet isolationn

Mouse and rat islets were isolated using the collagenase digestion method. In brief, 0.5 mg/mL collagenase dissolved in Hanks’ balanced salt solution was injected through the bile duct into the pancreas. Then, the pancreases were manually isolated and incubated in Krebs-Ringer bicarbonate buffer (KRBB) at 37 °C for 30 min. After homogenizing the pancreas with KRBB, the islets were separated by centrifugation in Ficoll gradient. Separated islets were resuspended in KRBB on a dish and handpicked under a light microscope for the experiments that followed.

### Cell lines

To examine glucose-stimulated and exendin-4-enhanced insulin secretion by overexpression or knock down of SKIP, a rat insulinoma cell line, INS-1D cells, was maintained in RPMI 1640 medium containing 11.1 mM glucose (Invitrogen) supplemented with 10% heat-inactivated FBS, 10 mM Hepes, 1 mM sodium pyruvate, 23.8 mM sodium bicarbonate, 50 *μ*M 2-mercaptoethanol, 100 IU/ml penicillin, and 100 *μ*g/ml streptomycin at 37 °C in a humidified atmosphere with 5% CO_2_ and 95% air.

### RT-PCR

Primers of target molecules were designed as follows. Mouse SKIP forward primer: 5′-ACCATGGATGTCAACTCCCGGCTT; mouse SKIP reverse primer: 5′-TTCTCTGTGATGCAGGCATC; mouse GAPDH forward primer: 5′-AACTTTGGCATTGTGGAAGG; mouse GAPDH reverse primer: 5′-ACACATTGGGGGTAGGAACA; rat SKIP forward primer: 5′-ACCATGGATGTCAACTCCCGGCTT; rat SKIP reverse primer: 5′-AGCTTCAACACGCTGGTCTC; rat GAPDH forward primer: 5′-AGACAGCCGCATCTTCTTGT; rat GAPDH Reverse primer: 5′-CTTGCCGTGGGTAGAGTCAT.

### Quantitative Real Time-PCR

Primers of target molecules were designed as follows. Rat SKIP forward primer: 5′-AGCTGGGCATCCCAACAATC; rat SKIP reverse primer: 5′-ATCCTAGTTCCAGGAGCCAGTCAA; rat Rps18 Forward primer: 5′-AAGTTTCAGCACATCCTGCGAGTA; rat Rps18 Reverse primer: 5′-TTGGTGAGGTCAATGTCTGCTTTC; mouse SKIP forward primer: 5′-ACCATGGATGTCAACTCCCGGCTT; mouse SKIP reverse primer: 5′-GTTTTCTGACTCATCTTCCACAAAC; mouse Rps18 Forward primer: 5′-CCAGTGGTCTTGGTGTGCTGA; Mouse Rps18 Reverse primer: 5′-TTCTGGCCAACGGTCTAGACAAC; mCherry forward primer: 5′-CCTGTCCCCTCAGTTCATGT; mCherry reverse primer: 5′-CCCATGGTCTTCTTCTGCAT. For gene typing primer, forward primer: 5′-GTAGAGGACAAATAGAGGGTCTTCA; reverse primer: 5′-GGAGTTTGAGAGACATCACATTAGG.

### Western blot

Tissue and cells protein was extracted by homogenization in lysis buffer (Sigma). Twenty micrograms of total protein were resolved by SDS-PAGE on 4–12% acrylamide gels (Invitrogen) and transferred to PVDF membranes (Invitrogen), followed by immunoblotting with antibody. Quantification of bands on western blot was accomplished by scanning the blots, then determining the densities of the bands using ImageJ software (National Institutes of Health, Bethesda, Maryland, USA).

### Plasmid preparation and transfection

The forward and reverse primer sequences for rat full-length SKIP were 5′-ACCATGGATGTCAA CTCCCGGCTT and 3′-TCCTAGTTCCAGGAGCCAGTCAAA, respectively. PCR products were inserted into pEF6/V5-His vector by TA cloning. All plasmids were sequenced and no mutations were found. pEF6/V5-His SKIP was transferred into 1 × 10^5^ INS-1D cells by Lipofectamine LTX (Invitrogen) and the cells were then cultured with antibiotic-free complete medium for 48 h for the following experiments.

### Antibodies

The following antibodies were used for western blot and immunohistochemistry: guinea pig polyclonal to insulin (Abcam, ab7842); rabbit monoclonal [D16G10] to glucagon (Cell signaling, #8233); mouse monoclonal to GAPDH (Santa cruz, sc-32233); mouse monoclonal [1C51] to mCherry (Abcam, ab125096).

We generated rabbit polyclonal anti-rat SKIP (amino acid 355–369; EQGSNHRDHDATPNS) antibody (SCRUM, Japan) and anti-mouse SKIP (amino acid 248–264; KETTQEGWDYHKEKLHC) antibody (Sigma, Japan).

### Immunohistochemical analysis

Rehydrated paraffin sections from pancreas samples (12-week-old SKIP^+/+^ mice and SKIP^−/−^ mice) were incubated overnight at 4 °C with primary anti-mCherry antibody (1:250), anti-insulin antibody (1:100) and anti-glucagon antibody (1:200). Images of islets were obtained by confocal microscopy (LSM 510 META system- Carl Zeiss Co., Ltd., Jena, Germany) at 40x magnification.

### Insulin secretion

Isolated islets were washed by KRBB containing 2.8 mM glucose, 0.2% BSA, and 10 mM Hepes (pH 7.4) and preincubated 37 °C for 1 h in the buffer. Ten islets were handpicked and exposed to four batches with different glucose concentrations (2.8 mM, 5.5 mM, 11.1 mM or 16.7 mM glucose) in 1 mL of the buffer and incubated at 37 °C for 1 h with or without 3-O-Methyl-D-glucose (non-metabolic glucose, 3-OMG) (Catalog number 135–10413, Wako, Japan), 50 mM potassium chloride (KCl) (Nacalai, Japan), 10 nM exendin-4 (ex-4, Catalog number E7144, Sigma), 10 μM Epac-selective activator 8-(4-chlorophenylthio)-2′-O-methyladenosine-3′, 5′-cyclic monophosphate, acetoxymethyl ester(ESCA, Catalog number C051, Biolog Life Science Institute), 10 nM PKA specific inhibitor myristolated PKA inhibitor (PKI, Catalog number 77-409, Invitrogen) and 500 μM non-selective phosphodiesterases (PDEs) inhibitor 3-isobutyl-1-metylxantine (IBMX, Catalog number I5879, Sigma). Insulin concentration and insulin content were measured by radioimmunoassay (Aloka Accuflex g7000; Hitachi, Tokyo, Japan). The amount of insulin secretion was normalized by cellular insulin content. For insulin secretion in INS-1D cells, the cells were cultured on 24-well plates coated with 0.001% poly-L-ornithine for 48 h, and washed with KRBH composed of 140 mM NaCl, 3.6 mM KCl, 0.5 mM MgSO_4_, 5 mM NaH_2_PO_4_, 1.5 mM CaCl_2_, 2 mM NaHCO_3_, 0.1% BSA and 10 mM Hepes (pH 7.4) with 2 mM glucose. The cells were then preincubated at 37 °C for 30 min in KRBH with 2.0 mM glucose, and further incubated at 37 °C for 30 min in KRBH with 2.0 mM or 10.0 mM glucose with or without 10 nM ex-4. Insulin concentration and insulin content were measured by radioimmunoassay. The amount of insulin secretion was normalized by the protein content.

### Glucose tolerance test in mice

Twelve-week-old mice were used for intraperitoneal glucose tolerance test (IpGTT). Glucose was administered by intraperitoneal injection at the dose of 1 g/kg body weight and 2 g/kg body weight after 18 h of fasting. Blood samples were obtained at the following time intervals: 0 (fasting levels), 15, 30, 60, and 120 min. Ex-4 was administered 30 min prior to IpGTT. Blood glucose levels were measured by glucose oxidase method (Sanwa Kagaku Kenkyusho, Nagoya, Japan). Insulin was measured by insulin ELISA kit (Morinaga, Japan).

### Measurement of ATP content

Isolated islets were washed with KRBB containing 2.8 mM glucose, 0.2% BSA, and 10 mM Hepes (pH 7.4) and preincubated at 37 °C for 1 h in the buffer. Ten islets were then handpicked and exposed in two batches having different glucose concentrations (2.8 mM, 16.7 mM glucose) in the buffer, and incubated at 37 °C for 1 h. The amount of ATP was measured with GLOMAX20/20 luminometer (Promega) and normalized by the protein content.

### Measurement of cAMP content

Isolated islets were preincubated at 37 °C for 1 h in KRBB containing 2.8 mM glucose, 0.2% BSA, and 10 mM Hepes (pH 7.4). Twenty-five islets were handpicked and exposed in 2.8 mM glucose with or without 10 nM ex-4 and incubated at 37 °C for 1 h, or incubated at 16.7 mM glucose at 37 °C for 0 min, 15 min or 60 min. cAMP content in supernatant was measured by using Direct cAMP ELISE kit (Enabling Discovery in Life Science, ENZO). The amount of cAMP content was normalized by the number of islets.

### Sphingosine kinase (SPHK) activity

Isolated islets were preincubated at 37 °C for 1 h in KRBB containing 2.8 mM glucose, 0.2% BSA, and 10 mM Hepes (pH 7.4). The islets were then stimulated by 2.8 mM or 16.7 mM glucose for 1 h. Lysed islets were used to measure sphingosine kinase activity using sphingosine kinase activity assay kit (Echelon). The amount of SPHK activity was normalized by the protein content.

### Calcium influx

Isolated islets were loaded with 5 μm Fura-2AM (Dojindo, Japan) at 37 °C for 30 min, placed in a heat-controlled chamber on the stage of an inverted microscope kept at 37 °C, superfused with KRBB containing 2.8 mM glucose, 0.2% BSA, and 10 mM Hepes (pH 7.4), and subsequently exposed to the buffer containing 16.7 mM glucose and then to buffer containing 2.8 mM glucose with 30 mM KCl. The islets were excited successively at 340 and 380 nm, and the fluorescence emitted at 510 nm was captured by an Olympus IX-70 microscope coupled to an ImagEM camera (Hamamatsu Photonics, Hamamatsu, Japan). The images were analyzed with the AQUACOSMOS analyzing system (Hamamatsu Photonics). The 340 nm (F340) and 380 nm (F380) fluorescence signals were detected every 20 seconds, and ratios (F340/F380) were calculated.

### Incubator two-photon excitation microscopy

Isolated islets were cultured in RPMI 1640 with 11.1 mM glucose overnight, and observed under incubation using incubator two-photon excitation microscopy (Olympus LCV110-MPE) at 37 °C in a humidified atmosphere (5% CO_2_ and 95% air). Two-photon excitation was effected at 1040 nm, the mCherry fluorescence was measured at 575–650 nm, the GFP fluorescence was measured at 515–560 nm, and negative control fluorescence was measured at 460–495 nm. Digital images (512 × 512 pixels) were taken and analyzed using IMARIS software.

### Statistics

Statistical analysis was performed with JMP software (Japan)**;** all results are expressed as averages ± standard errors of the mean (SEM). Where appropriate, one way analysis of variance (ANOVA) with Tukey test or student t-test was used to calculate difference between groups. P-values < 0.05 were considered statistically significant*, P-values < 0.005 were considered extremely significant**.

## Electronic supplementary material

supplymental information
