# Supplementary material for: Sphingosine kinase 1-interacting protein is a novel regulator of glucose-stimulated insulin secretion
Source: Sci Rep. 2017 Apr 10;7:779. doi: 10.1038/s41598-017-00900-7 (PMC5429731; doi:10.1038/s41598-017-00900-7)
Supplement: Supplementary file 1 — supplymental information [file 41598_2017_900_MOESM1_ESM.pdf]

Sphingosine kinase 1-interacting protein is a novel regulator of glucose-stimulated insulin secretion

Yu Wang [1], Shin-ichi Harashima [1,*], Yanyan Liu [1], Ryota Usui [1], Nobuya Inagaki [1, *]

[1] Department of Diabetes, Endocrinology and Nutrition, Graduate School of Medicine, Kyoto University, 606-8507, Japan

[*] Equally corresponding to:

Nobuya Inagaki, M.D., Ph.D. and Shin-ichi Harashima, M.D., Ph.D.
Department of Diabetes, Endocrinology and Nutrition, Graduate School of Medicine, Kyoto University

54 Shogoin Kawahara-cho, Sakyo-ku, Kyoto, 606-8507, Japan
TEL: +81-75-751-3560 FAX: +81-75-751-4244
E-mail: Nobuya Inagaki: inagaki@kuhp.kyoto-u.ac.jp
Shin-ichi Harashima: harasima@kuhp.kyoto-u.ac.jp

Cell line	Relative ratio of SKIP expression to Rps18
HEK293	0.0002%
NCI-H716	0.0015%
PC12	0.0022%
INS-1D	18.7%

Supplemental Table 1. Expression of SKIP in cell lines detected by qRT-PCR.

mRNA expression of SKIP in HEK293, NCI-H716, PC12 and INS-1D cells. HEK293: human embryonic kidney cell lines; NCI-H716: human intestine adenocarcinoma cell line, which secretes GLP-1; HepG2: human hepatoma cell line; PC12: rat adrenal pheochromocytoma cell line; INS-1D: rat insulinoma cell line, which secrets insulin.

Relative ratio to 2.8 mM glucose in the same genotype mice	2.8 mM glucose	5.5 mM glucose	11.1 mM glucose	16.7 mM glucose
fold insulin secretion in $SKIP^{+/+}$ islets	1	1.03±0.30	3.17±0.22	7.27±0.47
fold insulin secretion in $SKIP^{-/-}$ islets	1	1.71±0.61	5.50±0.37 *	14.38±2.08 *

n=7-8 mice per group and 5-6 samples per group, with 10 islets per sample, *p<0.05, $SKIP^{+/+}$ vs $SKIP^{-/-}$.

Supplemental Table 2. Relative ratio to 2.8 mM glucose of GSIS in the same genotype mice islets.

Fold insulin secretion at 2.8 mM, 5.5 mM, 11.1 mM, and 16.7 mM glucose in $SKIP^{+/+}$ and $SKIP^{-/-}$ islets, respectively. n=7-8 mice per group and 5-6 samples per group, with 10 islets per sample, *p<0.05, $SKIP^{+/+}$ vs $SKIP^{-/-}$. Data are expressed as average ± standard error of the mean (SEM). Significance was determined by student's t-test.

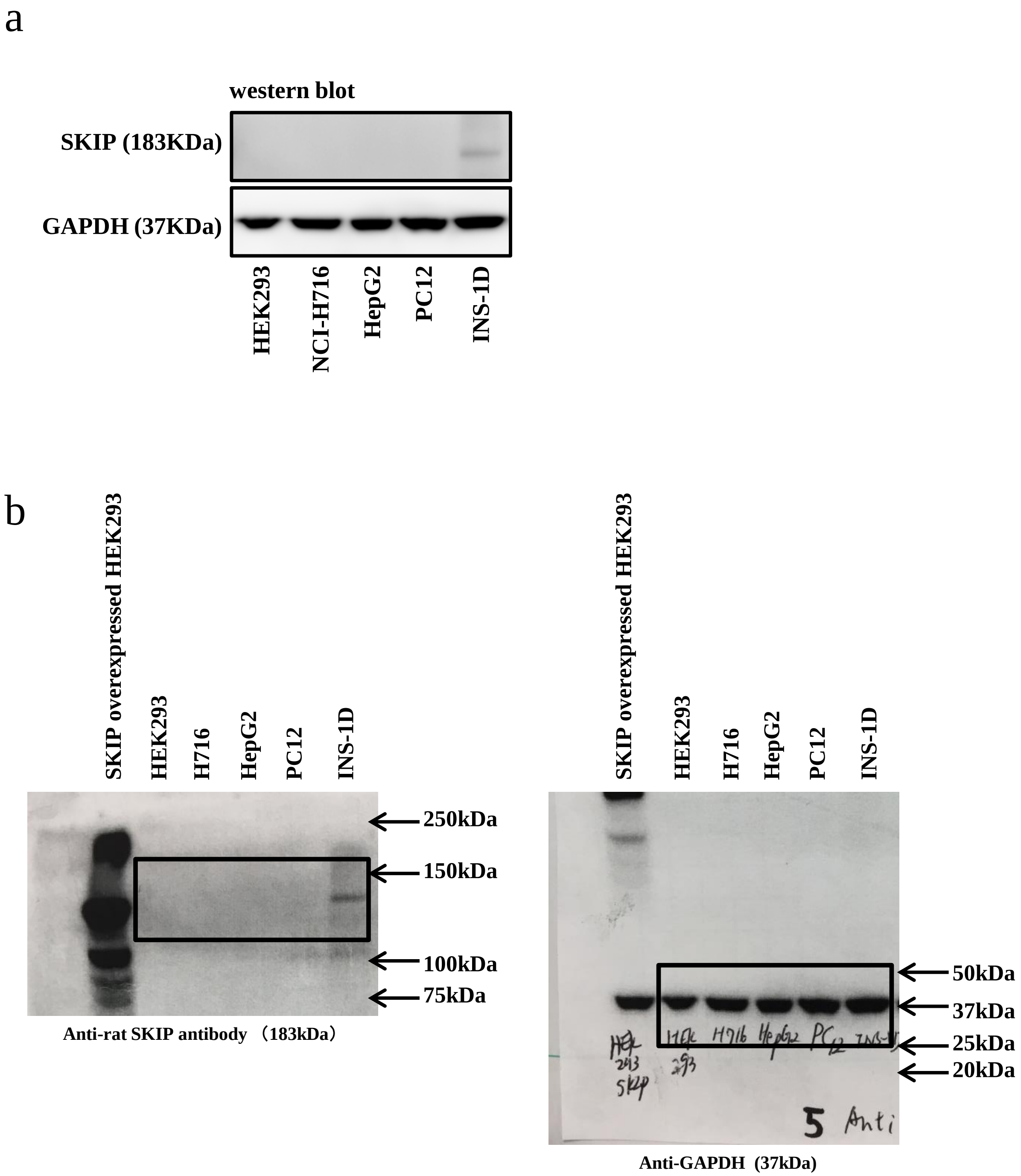

Supplemental Figure 1. Expression of SKIP in cell lines detected by western blot.

(a) Protein expression of SKIP in HEK293, NCI-H716, HepG2, PC12 and INS-1D cells with anti-rat SKIP antibody. The membrane was reprobed with anti-GAPDH antibody as control. (b) Original images of western blots. Boxes areas indicated the cropped regions. All the gels were run under the same experimental conditions.

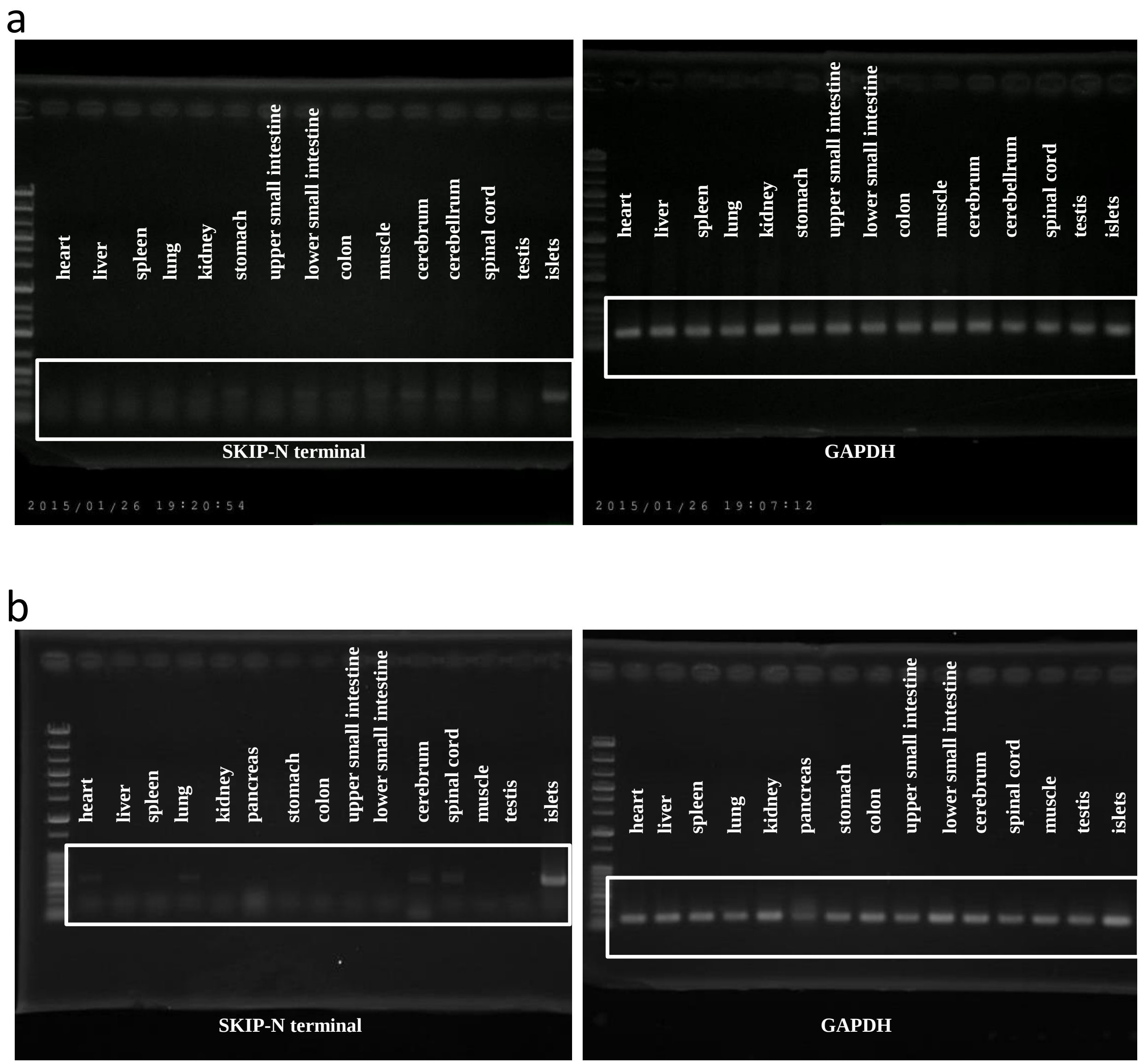

Supplemental Figure 2. Uncropped images of RT-PCR gels shown in Figure 1.

(a) Uncropped images of Figure 1a. (b) Uncropped images of Figure 1b. Original images of RT-PCR. Boxes areas indicated the cropped regions.

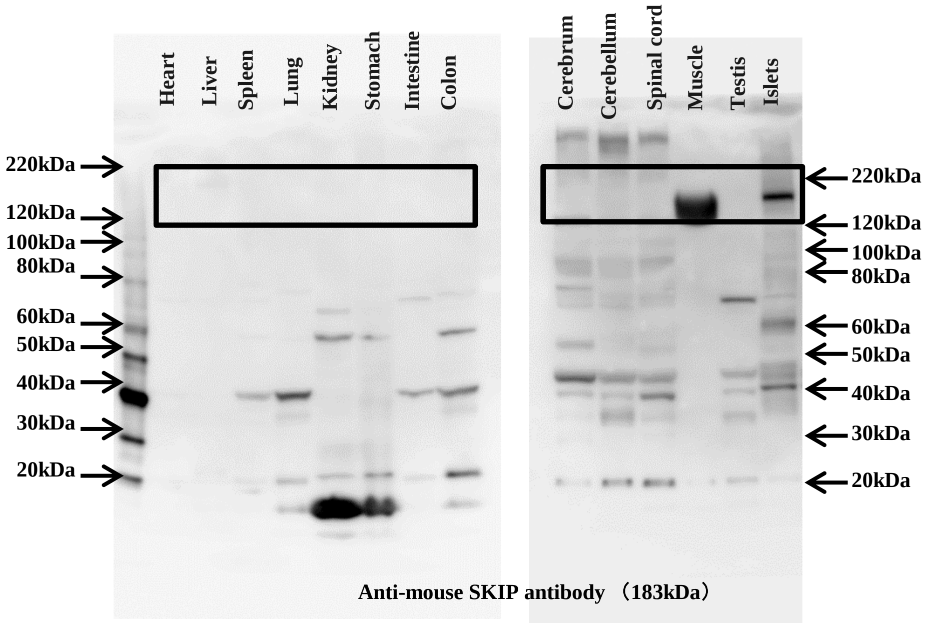

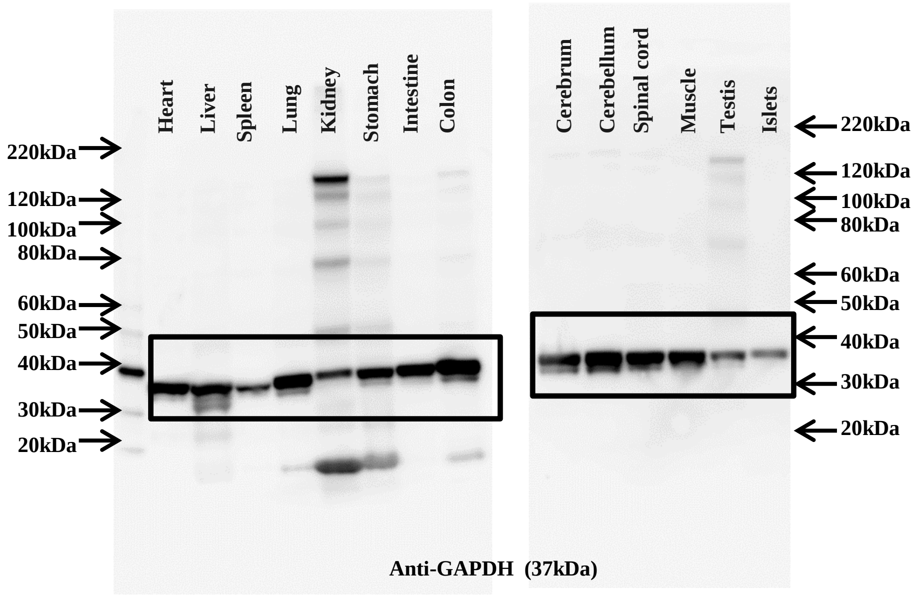

Supplemental Figure 3. Uncropped images of western blots shown in Figure 1c.

Original images of western blots. Boxes areas indicated the cropped regions.

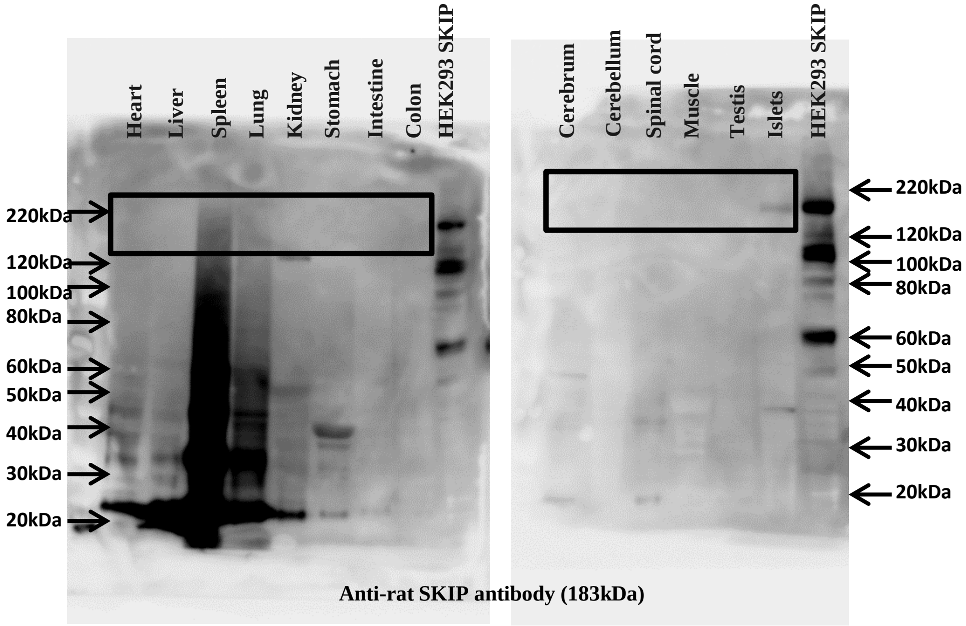

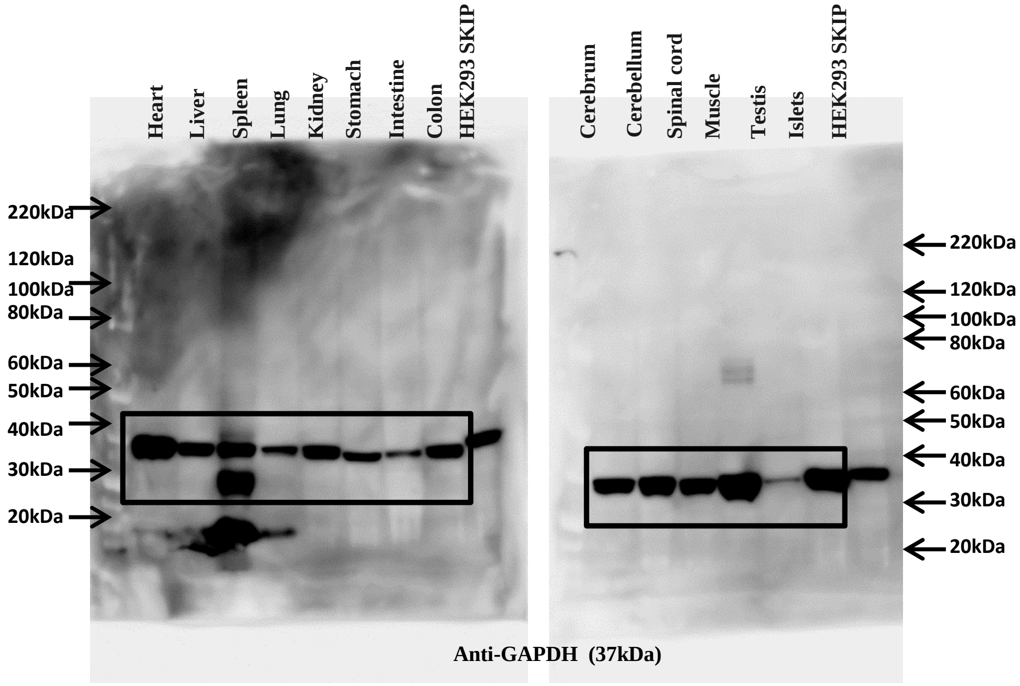

Supplemental Figure 4. Uncropped images of western blots shown in Figure 1d.

Original images of western blots. Boxes areas indicated the cropped regions.

a

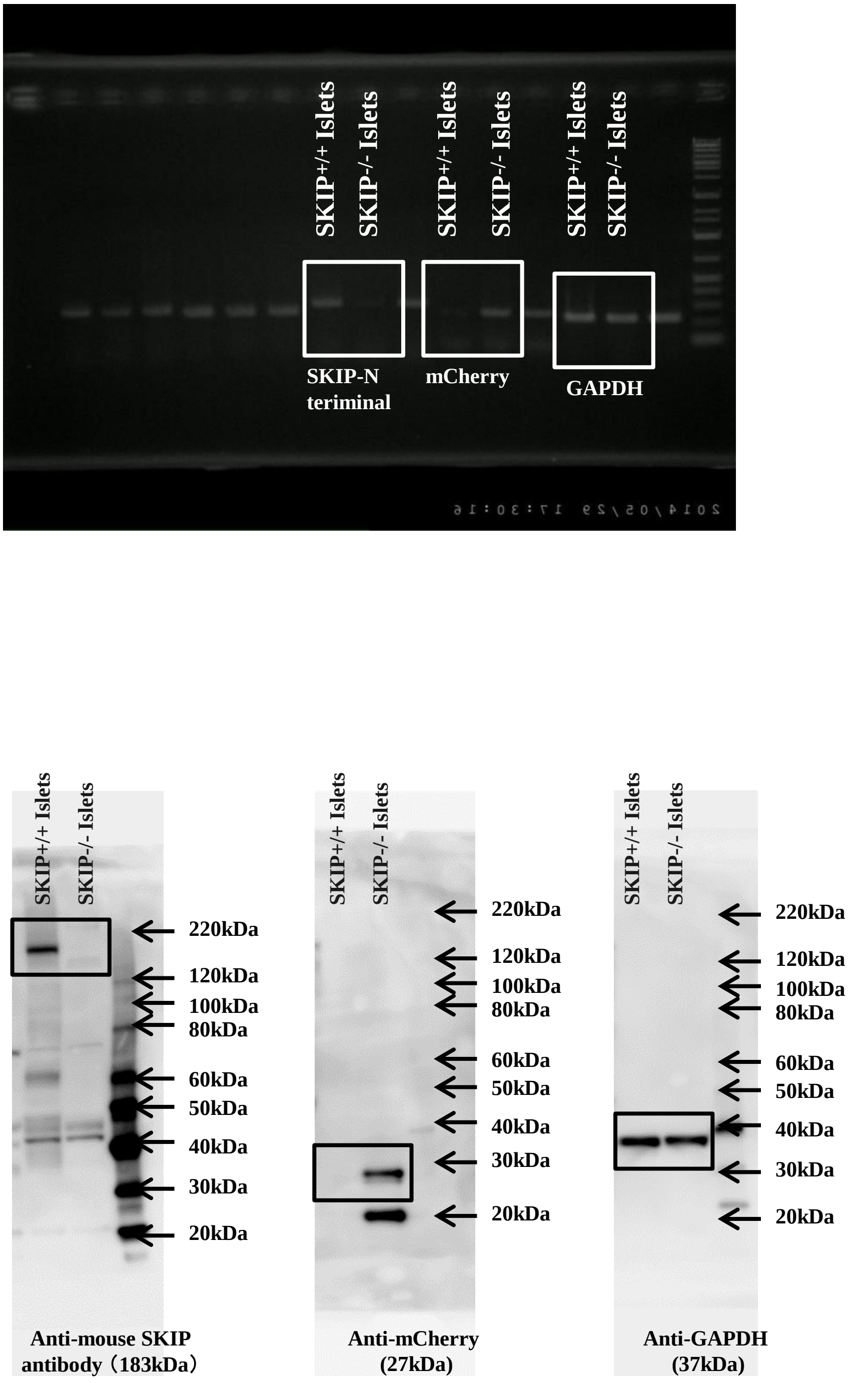

Supplemental Figure 5. Uncropped images of RT-PCR gel and western blots shown in Figure 2.

(a) Original images of RT-PCR gel. Boxes areas indicated the cropped regions.
(b) Original images of western blots. Boxes areas indicated the cropped regions.

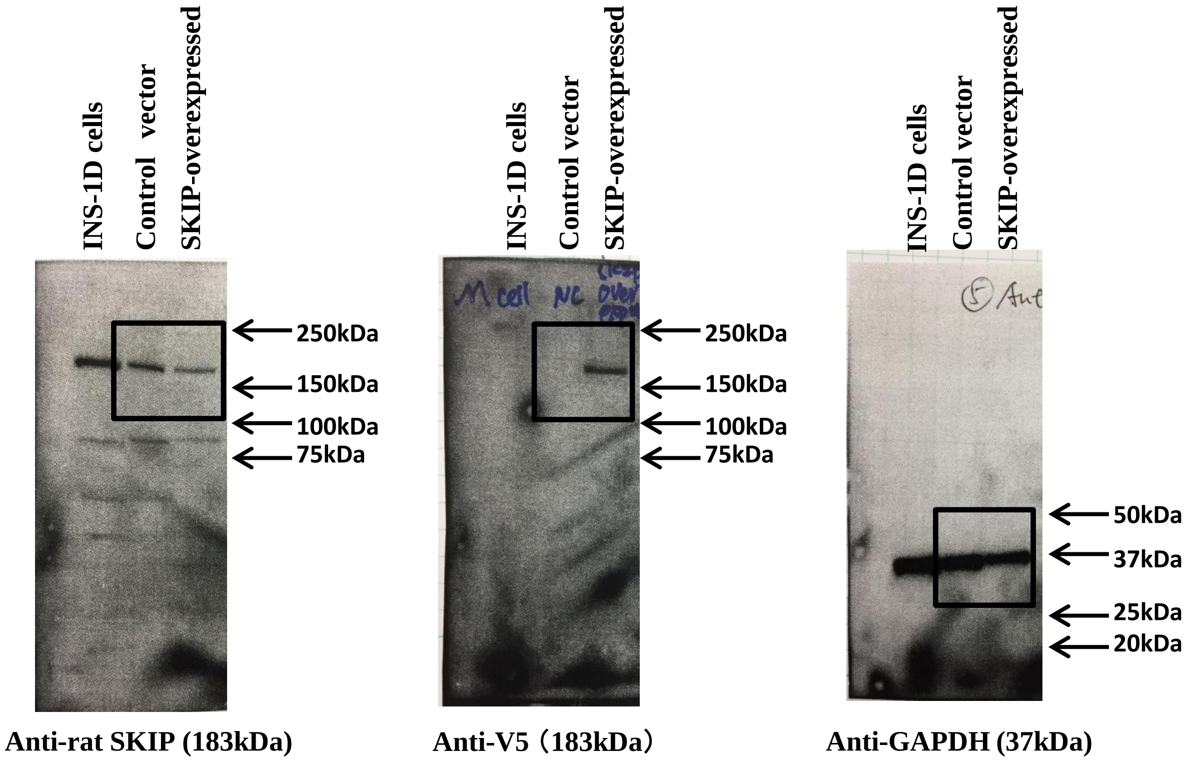

Supplemental Figure 6. Uncropped images of RT-PCR gel and western blots shown in Figure 5.

Original images of western blots. Boxes areas indicated the cropped regions.
